# Mitochondrial dysfunction and epithelial to mesenchymal transition in head neck cancer cell lines

**DOI:** 10.1038/s41598-022-16829-5

**Published:** 2022-08-02

**Authors:** Maria Carmo Greier, Annette Runge, Jozsef Dudas, Viktoria Pider, Ira-Ida Skvortsova, Dragana Savic, Herbert Riechelmann

**Affiliations:** 1 Department of Otorhinolaryngology, Head and Neck Surgery, Medical University of Innsbruck, Innsbruck, Austria; 2 Department of Therapeutic Radiology and Oncology, Medical University of Innsbruck, Innsbruck, Austria; 3 EXTRO-Lab, Tyrolean Cancer Research Institute, Department of Therapeutic Radiology and Oncology, Medical University of Innsbruck, Innsbruck, Austria

**Keywords:** Head and neck cancer, Cancer, Metabolic disorders, Cellular imaging, Mitochondria, Energy metabolism, Cell migration, Epithelial-mesenchymal transition, Oncology, Cancer

## Abstract

Mitochondrial dysfunction promotes cancer aggressiveness, metastasis, and resistance to therapy. Similar traits are associated with epithelial mesenchymal transition (EMT). We questioned whether mitochondrial dysfunction induces EMT in head and neck cancer (HNC) cell lines. We induced mitochondrial dysfunction in four HNC cell lines with carbonyl cyanide-4(trifluoromethoxy)phenylhydrazone (FCCP), a mitochondrial electron transport chain uncoupling agent, and oligomycin, a mitochondrial ATP synthase inhibitor. Extracellular flux analyses and expression of the cystine/glutamate antiporter system xc (xCT) served to confirm mitochondrial dysfunction. Expression of the EMT-related transcription factor SNAI2, the mesenchymal marker vimentin and vimentin/cytokeratin double positivity served to detect EMT. In addition, holotomographic microscopy was used to search for morphological features of EMT. Extracellular flux analysis and xCT expression confirmed that FCCP/oligomycin induced mitochondrial dysfunction in all cell lines. Across the four cell lines, mitochondrial dysfunction resulted in an increase in relative SNAI2 expression from 8.5 ± 0.8 to 12.0 ± 1.1 (mean ± SEM; p = 0.007). This effect was predominantly caused by the CAL 27 cell line (increase from 2.2 ± 0.4 to 5.5 ± 1.0; p < 0.001). Similarly, only in CAL 27 cells vimentin expression increased from 2.2 ± 0.5 × 10^–3^ to 33.2 ± 10.2 × 10^–3^ (p = 0.002) and vimentin/cytokeratin double positive cells increased from 34.7 ± 5.1 to 67.5 ± 9.8% (p = 0.003), while the other 3 cell lines did not respond with EMT (all p > 0.1). Across all cell lines, FCCP/oligomycin had no effect on EMT characteristics in holotomographic microscopy. Mitochondrial dysfunction induced EMT in 1 of 4 HNC cell lines. Given the heterogeneity of HNC, mitochondrial dysfunction may be sporadically induced by EMT, but EMT does not explain the tumor promoting effects of mitochondrial dysfunction in general.

## Introduction

In cancer cells, mitochondrial structure and function are altered, resulting in mitochondrial dysfunction^1^. Mitochondrial dysfunction impairs cellular energy production, increases glycolysis and acidosis, and leads to deregulation of cellular metabolism, a hallmark of cancer^2–5^. This process is associated with cancer aggressiveness, invasion, metastasis, and drug resistance^6,7^. These traits are also characteristic of epithelial to mesenchymal transition (EMT)^8–10^, a complex, variable, and context-specific cellular program that enables carcinoma cells to suppress epithelial characteristics in favour of mesenchymal differentiation^11^. In cancer, EMT is usually incomplete and reversible, resulting in coexpression of epithelial and mesenchymal markers^12^. EMT-associated reprogramming is regulated by various transcription factors, including SNAI1, SNAI2, ZEB, and TWIST^13,14^. In head and neck cancer, SNAI2 plays a dominant role^13,15^. Among others, the transforming growth factor beta (TGF-β1) regulatory pathway is a canonical trigger of EMT^16,17^.

Mitochondrial dysfunction and EMT regulatory pathways are interrelated. Mitochondrial dysfunction can activate glycolytic pathways, which interact with EMT-related transcription factors such as Snail. Snail represses enzymes of the glycolytic pathway as well as enzymes of the gluconeogenesis pathway, such as fructose-1,6-bisphosphatase, which leads to enhanced glycolytic flux^18^. Downregulation of the expression of mitochondrial proteins involved in oxidative phosphorylation (OXPHOS) was associated with EMT gene signatures and enhancement of EMT^7,19^. Induction of mitochondrial dysfunction via oligomycin A and antimycin A resulted in downregulation of E-cadherin expression, upregulation of vimentin expression, and increased migration and invasion, suggesting activation of the EMT cellular program^20^. Although these studies were not performed in head and neck cancer, they suggest a relationship between mitochondrial dysfunction and EMT.

Head and neck squamous cell carcinoma (HNSCC) is a common malignancy derived from mucosal surfaces of the oral cavity, sinonasal cavity, pharynx, and larynx. HPV-induced HNSCC differs substantially from HPV-negative HNSCC; however, both entities show high inter- and intratumoral tumour cell heterogeneity^21,22^. In this study, we chose 4 different HPV-negative HNSCC cell lines for the experiments. Our aim was to examine whether induced mitochondrial dysfunction leads to EMT in different HPV-negative HNSCC cell lines. Mitochondrial dysfunction was induced by blocking mitochondrial OXPHOS and measured using extracellular flux analysis and expression of the cystine/glutamate transporter xCT (SLC7A11)^23^. In extracellular flux analysis, the oxygen consumption rate (OCR) serves as a measure of OXPHOS, and the extracellular acidification rate (ECAR) serves as a measure of glycolysis. A high OCR/ECAR ratio indicates a metabolic state driven by OXPHOS. Solute carrier family 7 member 11 (SLC7A11) is the coding gene for xCT, which is known to be overexpressed in cells with mitochondrial damage with low OXPHOS^24^. The OCR/ECAR ratio and xCT expression are inversely correlated. EMT was assessed by gene expression analysis of SNAI2 and vimentin, flow cytometric analysis of vimentin/cytokeratin double-positive cells, and holotomographic microscopy^12^.

## Methods

### Cell lines

The 4 HNSCC cell lines SCC-25^23^, UPCI-SCC-003^25^, HN^26^ and CAL 27^27^ were purchased from the German Collection of Microorganisms DSMZ, Braunschweig, Germany. Cells were thawed at 37 °C in 8 mL of medium in 15 mL Falcon tubes, centrifuged for 5 min at 290*g*/at 4 °C and cultivated in a 1:1 mixture of Dulbecco's modified Eagle's medium and Ham’s F12 with additional 1% penicillin/streptomycin, 1% MEM with nonessential amino acids (NEAAs), 1 mM sodium pyruvate 100 mM (all from PAN-Biotech, Aidenbach, Germany), 10% foetal bovine serum and 1% l-glutamine 200 mM (both from Gibco, Grand Island, NY, USA). The cells were stabilized by two passages and cultured for 2 weeks prior to the experiments at 5% CO_2_ and 37 °C.

### Induction of mitochondrial dysfunction

For induction of mitochondrial dysfunction, a mixture of carbonyl cyanide-4(trifluoromethoxy) phenylhydrazone (FCCP) and oligomycin was used (#103275-100, Agilent Bioscience, Sta. Clara, USA)^28,29^ or prepared analogously as described in Supplement 3. Cells were plated in XFp Cell Culture Miniplates (#103022-100, Agilent Bioscience, Sta. Clara, USA) in DMEM/F12 (0.3%) at a density of 2 × 10^4^ cells per mL, which created a confluence of approximately 70%. For TGF-β1 controls, 1 μL/mL TGF-β1 (#P01137, R&D Systems, Minneapolis, USA) was added to the cell culture medium, and the cells were incubated overnight (5% CO_2_/37 °C) prior to the addition of FCCP/oligomycin.

### Assessment of mitochondrial dysfunction

Extracellular flux analysis^29^ with the Seahorse XFp Analyzer (Agilent Bioscience, Sta. Clara, USA) and cystine/glutamate transporter (xCT) expression served to confirm mitochondrial dysfunction. The FCCP/oligomycin mixture (XFp Cell Energy Phenotype Stress Kit; #103275-100, Agilent Bioscience, Sta. Clara, USA) was assembled as described in the manufacturer’s protocol and added immediately before the measurement. The Seahorse XFp Analyzer was used for measurement 4× during baseline and 4× during FCCP/oligomycin conditions in a 5 min interval (Supplement 1). Parameters were normalized to 10^4^ cells/well (Supplement 2).

For cystine/glutamate transporter (xCT) expression, 2 × 10^5^ cells/mL were plated in four petri dishes in 10 mL of DMEM/F12 (0.3% FBS) each, two of them with an additional 1 ng/mL TGF-β1 (positive control for EMT). After 24 h, two dishes (one with TGF-β1 and one without) were exposed to 1 µM FCCP/oligomycin (Supplement 3) for 50 min in a CO_2_-free incubator at 37 °C to mimic the XFp Seahorse measurement. The other two dishes were incubated the same way without FCCP/oligomycin.

After FCCP/oligomycin treatments, cells were cultivated for one additional day without any further treatments. On this day, cells were allowed to adapt and react prior to RNA and protein isolation. For RNA and protein measurement, cells were lysed as described in the “RNA isolation, reverse transcription and RT-PCR” section below. For qPCR, the primer sequences for xCT (SLC7A11) were downloaded from the PrimerBank of the Massachusetts General Hospital, Boston, MA, USA^28^. Primer sequences are listed in Suppl. Table 1. Primers were synthesized by Invitrogen (Darmstadt, Germany) and were used together with the Sensifast Sybr Fluorescein Kit of Bioline (Labconsulting, Vienna, Austria) in a Bio-Rad MyiQ™ (Bio-Rad, Laboratories, Inc., Hercules, CA, USA) cycler according to the manufacturer's protocol. GAPDH was used as a housekeeping gene, and the relative quantities of SLC7A11 were calculated as described above.

### Assessment of epithelial mesenchymal transition (EMT)

EMT was assessed by gene expression of vimentin and SNAI2 using quantitative PCR and by vimentin/cytokeratin coexpression with flow cytometry in the FCCP/oligomycin-treated cells (see FCCP/oligomycin treatment above). In addition, all cell lines were examined under a holotomographic microscope to detect EMT-typical morphological changes. Primer sequences for vimentin and SNAI2 were downloaded from PrimerBank at Massachusetts General Hospital, Boston, MA, USA (Suppl. Table 1). After FCCP/oligomycin treatment and one additional cultivation day, the cells were lysed, and RT-PCR was performed as described for xCT. GAPDH was used as a housekeeping gene, and the relative amounts of vimentin and SNAI2 transcripts were calculated as described above.

The percent of vimentin-cytokeratin double-positive cells was determined in a CytoFLEX flow cytometer (Beckman Coulter). Therefore, 2 × 10^5^ cells/ml were plated in Petri dishes with 10 ml of DMEM/F12 (0.3% FBS) and treated as described for xCT analyses. After FCCP/oligomycin treatment and an extra cultivation day to allow the cells to respond, the cells were collected by trypsinization, counted and suspended in FBS (#26140087, Gibco, MA, USA). Then, the cell suspensions were incubated with antibodies against cytokeratin and vimentin (Suppl. Table 2) for 20 min at room temperature using the Perfix NC kit from Beckman Coulter (Marseille, France) according to the manufacturer's instructions. One reaction was set for 2 × 10^6^ cells. Negative event gates were set by the isotype controls containing 99% of the isotype events. Positive events were defined as events with higher signals than the negative control gates. At least 2 replicates were analysed, and all samples were measured in duplicate.

**Figure 1:**
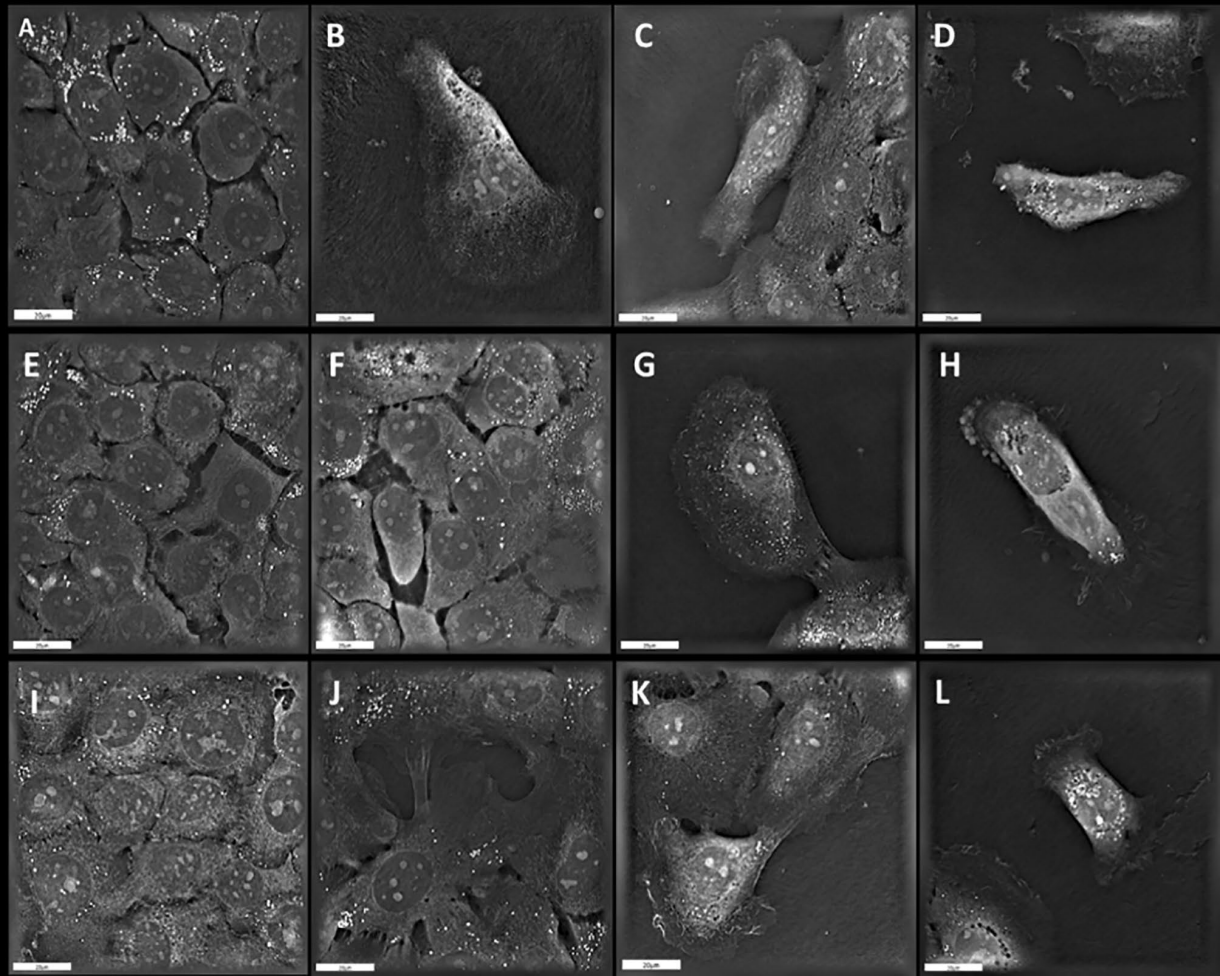
Holotomographic microscopy scoring example of EMT characteristics (SCC25). (**A**) Score 0 for fibroblast-like-morphology (FM). (**B**) Score 1 for FM. (**C**) Score 2 for FM and (**D**) score 3 for FM. (**E**) Score 0 for cell individualization. (**F**) Score 1 for cell individualization. (**G**) Score 2 for cell individualization and (**H**) score 3 for cell individualization. (**I**) Score 0 for surface adhesion, (**J**) Score 1 for surface adhesion. (**K**) Score 2 for surface adhesion and (**L**) Score 3 for surface adhesion. Live cell imager (Nanolive, Switzerland); 20 μm.

For holotomographic microscopy, the same conditions and media were used as for extracellular flux analysis. The only difference was that 2 × 10^5^ cells/mL were plated in IbiDi dishes (IbiDi, Ltd., Planegg, Germany) instead of Agilent XFp cell culture miniplates, and FCCP/oligomycin was added manually. After the same treatment time as for the flux analyses in a CO_2_-free incubator, the XFp measurement medium was replaced with DMEM/F12 (0.3% FBS), and the cells were immediately analysed under a 3D Cell Explorer Holotomography microscope (Nanolive SA, Switzerland) with an air objective at 60× magnification. Three typical histomorphologic features of EMT were assessed and semiquantitatively scored from 0 to 3: fibroblast-like morphology (FM), cell individualization, and cell detachment. For these 3 features, a score of 0 indicates 0–10% of cells have this feature; 1 indicates 11–50%; 2 indicates 51–80%; and 3 indicates ≥ 80% of cells have this feature (Fig. 1).

### RNA isolation, reverse transcription and RT-PCR

For gene expression analyses, cells were lysed in 1 mL of TRIzol^®^ Reagent (Ambion^®^, Life TechnologiesTM, Carlsbad, CA, USA), and RNA was isolated following the instructions of the manufacturer. RNA concentrations were determined by absorption at 260 nm and fluorometric measurements (Qubit, Invitrogen, Darmstadt, Germany), and RNA quality and integrity were evaluated by a Qubit RNA IQ kit (Invitrogen). The proportion of intact RNA in the total RNA isolates was at least 70%. Two micrograms of total RNA was reverse transcribed by M-MuLV Reverse Transcriptase with 2 µg of oligo dT15 (GeneON, Ludwigshafen am Rhein, Germany) in a ThermoQ heating and cooling block (Biozym, Hessisch Oldendorf, Germany). cDNA samples representing 10 ng of original total RNA were subjected to real-time qPCR. GAPDH was used as a housekeeping gene, and relative quantities of the transcripts were calculated by pairwise differences of threshold cycles (δCT) of the gene of interest and the loading control housekeeping gene^30^. For the final analysis, we used the relative quantification and related the PCR signal in the cells to a control reference gene expression level^12^. The identity of the PCR products of genes was confirmed by Sanger sequencing by Microsynth Austria (Vienna, Austria)^12^.

### Data analysis

The full factorial experimental design with 2 (with/without FCCP/oligomycin) × 2 (with/without TGF-ß1) × 4 (cell lines) × 4 replicates = 64 experiments was analysed with a generalized linear model. The results of PCR, flow cytometry, and extracellular flux analysis revealed a right-skewed distribution. Therefore, a gamma distribution with a log linkage function was modelled. The estimated marginal means (EMM) and their standard errors (SEM) are reported. Holotomography scores for EMT-typical cellular responses, ranging from 0 to 9, were compared with and without FCCP/oligomycin using the Fisher-Freeman-Halton exact test. Calculations were performed with SPSS Ver. 27 (IBM, Armonk, NY).

## Results

### Effect of FCCP/oligomycin on EMT

**Figure 2:**
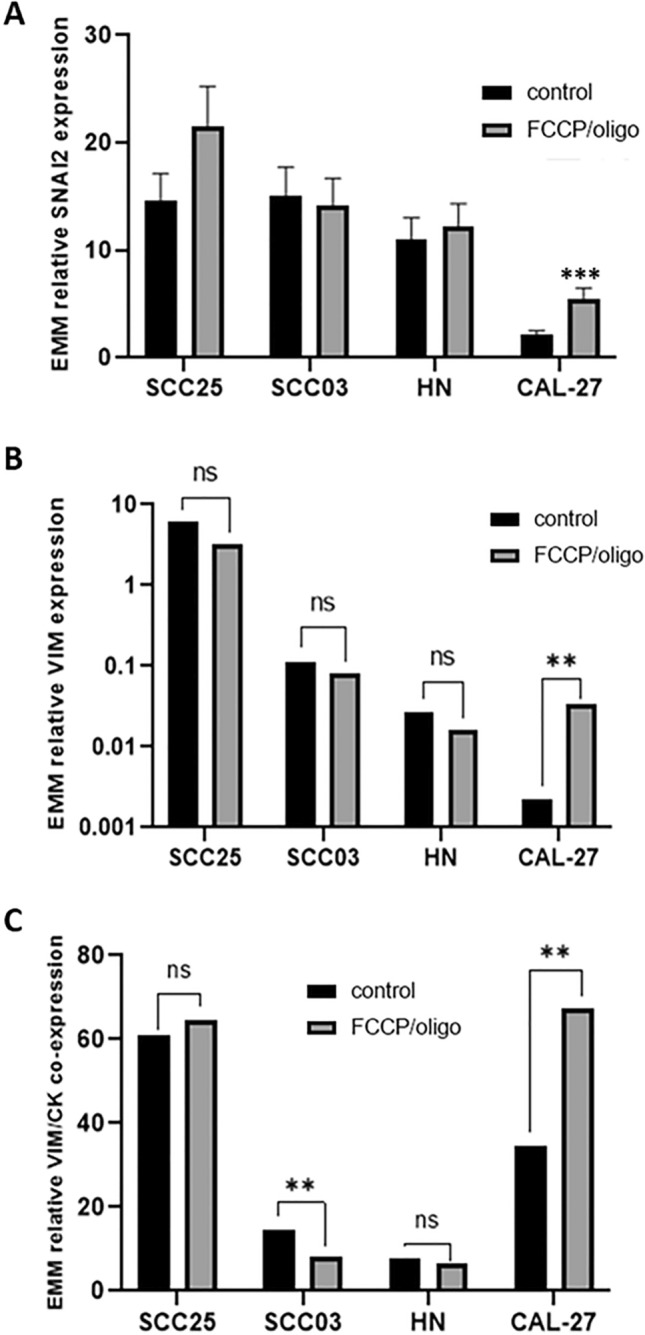
Relative SNAI2 (**A**), vimentin (VIM; **B**) expression, and percentage of vimentin/cytokeratin double positive cells (**C**) in response to FCCP/oligomycin (EMM: Estimated marginal mean; Bars: SEM; *p < 0.05, **p < 0.01, ***p < 0.001).

**Figure 3:**
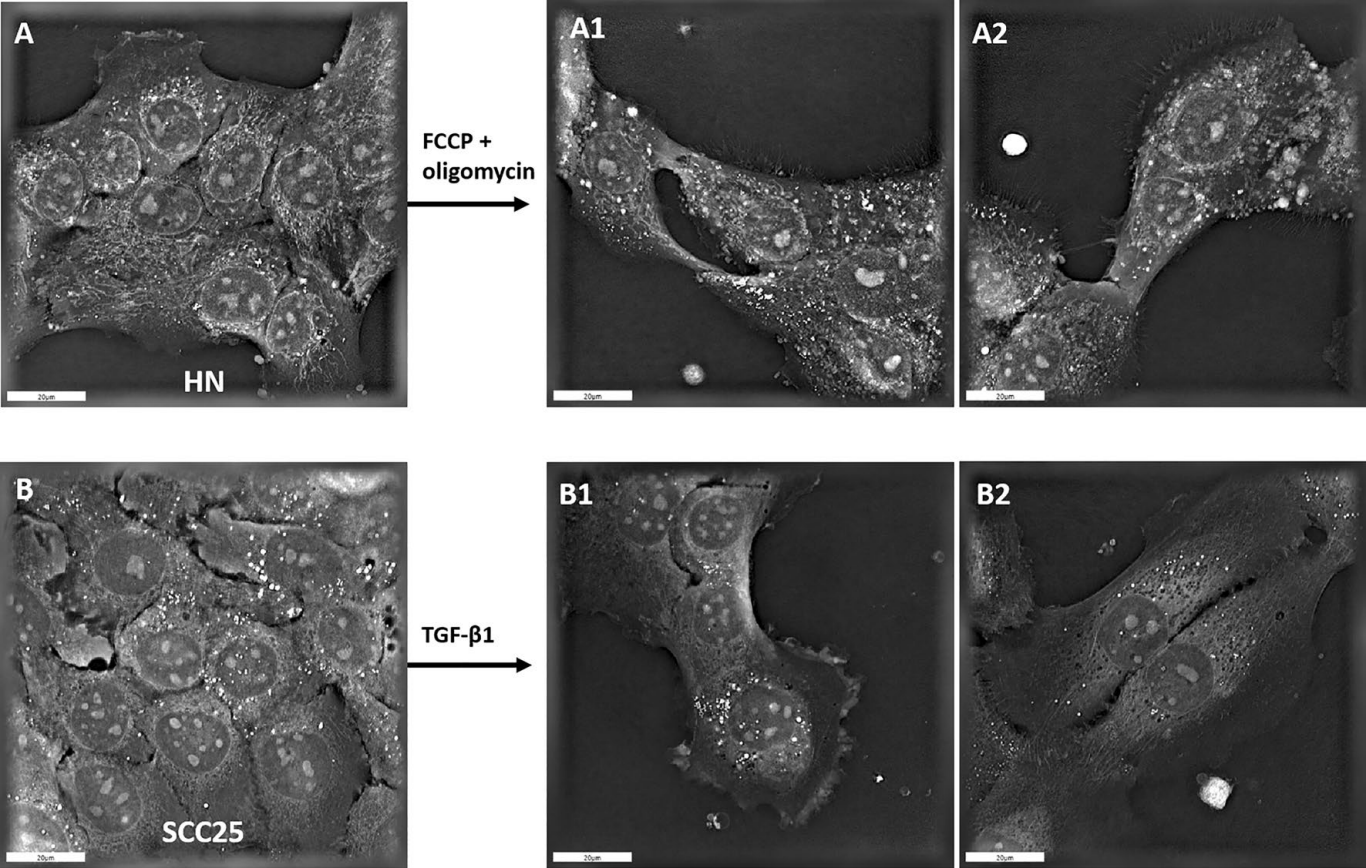
Holotomographic microscopy of HN cells (**A**) in response to FCCP/oligomycin addition (A1 + A2) and SCC25 cells (**B**) in response to TGF-ß1 (B1 + B2). Bars: 20 μm.

The effects of FCCP/oligomycin on EMT depended on the experimental cell line. Considering all four cell lines together, the addition of FCCP/oligomycin increased the relative expression of SNAI2 from 8.5 ± 0.8 to 12.0 ± 1.1 (p = 0.007). When we separately examined the effect of FCCP/oligomycin on each cell line, only CAL 27 cells showed increased SNAI2 expression (p < 0.001; Fig. 2A), while the effect was not significant in the other 3 cell lines (all p > 0.1). The addition of FCCP/oligomycin had no effect on the overall relative expression of vimentin (p = 0.137). For the effect of FCCP/oligomycin on vimentin expression in individual cell lines, an increase was observed for CAL 27 cells from 2.2 ± 0.5 × 10^–3^ to 33.2 ± 10.2 × 10^–3^ (p = 0.002; Fig. 2B). Finally, the percentage of vimentin-cytokeratin double-positive cells in flow cytometry did not change in the overall analysis of all cell lines; however, at the cell line level, it decreased in SCC03 cells (p = 0.009) and increased in CAL 27 cells (p = 0.003) following FCCP/oligomycin administration (Fig. 2C). Possible EMT scores in holotomographic microscopy ranged from 0 (no EMT) to 9. Holotomographic EMT scores in 32 experimental sets were mostly below five, indicating generally few cells with EMT-typical morphological changes (Table 1). In particular, no significant effects of FCCP/oligomycin on EMT-typical morphological cell characteristics were observed (Fischer-Freeman-Halton p = 0.9). Occasionally, FCCP/oligomycin induced EMT-typical changes in cell morphology (Fig. 3A).

**Table 1:** Count of cell lines with EMT-scores in holotomographic microscopy of 4 HNSCC cell lines (range of possible scores from 0 to 9) with and without FCCP/oligomycin (p = 0.9).

EMT Score	Count of cell lines without FCCP/oligomycin	Count of cell lines with FCCP/oligomycin	Total
2	3	3	6
3	6	8	14
4	6	5	11
5	1	0	1
Total	16	16	32

### Cell line heterogeneity

Consistent with the heterogeneity of head and neck cancer, the four HNSCC cell lines used differed markedly in all parameters investigated (Fig. 2A–C, control). The baseline expression of SNAI2 differed by up to fivefold among the four cell lines (p < 0.001; Fig. 2A) SCC25 cells had a baseline relative SNAI2 expression of 17.7 ± 2.2, and CAL 27 cells had a baseline relative expression of 3.5 ± 0.4. Marked differences between cell lines were also observed for baseline vimentin expression (p < 0.001). Again, SCC25 cells had the highest expression at 4.7 ± 1.1, and CAL 27 cells had the lowest expression at 2.2 ± 0.5 × 10^–3^ (Fig. 2B). Flow cytometry results also yielded different baseline percentages of vimentin/cytokeratin double positivity in the four cell lines (p < 0.001; Fig. 2C). Here, SCC25 and CAL 27 cells had the highest values. SCC03 and HN had similarly low values (Fig. 2C).

### Evidence of successful induction of mitochondrial dysfunction

**Figure 4:**
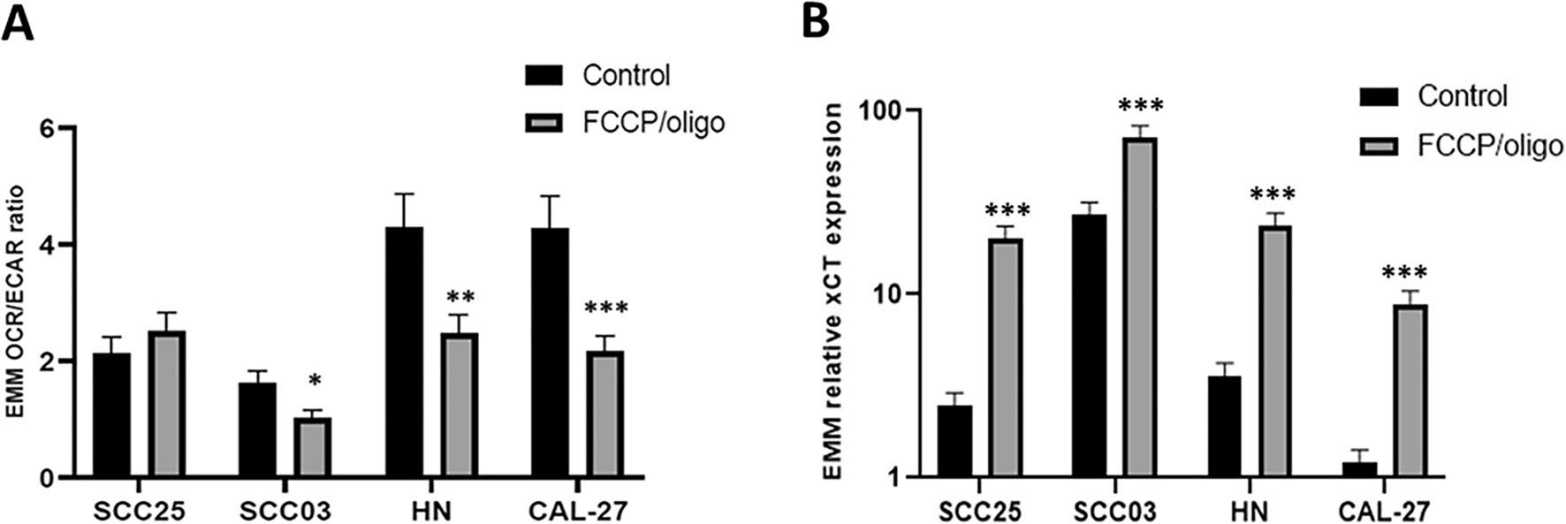
OCR/ECAR ratio (**A**) and xCT expression (**B**) of 4 HNSCC cell lines in response to FCCP/oligomycin (EMM: Estimated marginal mean; bars: SEM; *p < 0.05, **p < 0.01, ***p < 0.001).

A high OCR/ECAR ratio indicates a metabolic phenotype driven by OXPHOS, whereas lower OCR/ECAR ratios indicate glycolysis due to mitochondrial dysfunction. The observed decrease in the OCR/ECAR ratio confirmed FCCP/oligomycin-induced mitochondrial dysfunction in all cell lines except SCC25 cells (Fig. 4A; Suppl. Table 3). Moreover, FCCP/oligomycin addition increased xCT values in all cell lines (Fig. 4B; Suppl. Table 4). As with the markers for EMT, the baseline OCR/ECAR ratio (p < 0.001) and baseline xCT expression (p < 0.001) revealed significant differences between the cell lines studied (Suppl. Fig. 1).

### Evidence that cell lines were EMT-competent

TGF-ß1 is a key mediator of EMT in cancer cells; however, not all HNSCC cell lines are capable of EMT. The responses of SNAI2 and vimentin to TGF-ß1 stimulation indicate that the four cell lines used can respond to an appropriate stimulus with EMT (Suppl. Fig. 2; Suppl. Table 5). Double positivity of vimentin and cytokeratin in flow cytometry did not significantly increase after TGF-ß1 stimulation (Suppl. Fig. 2, Suppl. Table 5), but a trend towards EMT-like histomorphological features was observed by holotomographic microscopy (p = 0.1; Fig. 3; Suppl. Table 6).

## Discussion

EMT contributes to the aggressiveness and therapeutic resistance of head and neck carcinomas^8^. In addition to genetic reprogramming and external stimuli from the tumour microenvironment^31^, there is ample evidence that different kinds of cellular stress promote EMT in carcinomas^32,33^. Here, we investigated cellular stress induced by mitochondrial dysfunction, a particular form of cellular stress that is common in HNC^2^. We questioned whether cellular stress due to mitochondrial dysfunction is a significant EMT trigger. In 4 HNC cell lines, mitochondrial dysfunction was induced by FCCP/oligomycin, an established method to inhibit mitochondrial OXPHOS. This change leads to a metabolic state dominated by glycolysis, as is the case with HNC in vivo.

Clinically, HNC exhibits considerable cellular heterogeneity. Hence, we used several HNC cell lines for this study. To reduce variability, we used only HPV-negative cell lines. Consequently, the results are not applicable to HPV-positive cell lines. Different HNC cell lines might prefer different regulatory pathways and/or have a heterogeneous bioenergetic organization^34,35^. Indeed, some cell lines investigated were constitutively more glycolytic, and others depended more on OXPHOS. We also observed high variability among EMT parameters in the 4 cell lines (Fig. 4). This finding may in part explain why the EMT response of the HNC cell lines to mitochondrial dysfunction in this study differed.

To determine whether mitochondrial dysfunction had actually been induced, we used extracellular flux analysis, a relatively precise method to measure metabolic activity and state in cells^29^. We also examined the expression of xCT, a reliable surrogate marker of mitochondrial dysfunction^24^. Both methods indicated the successful induction of mitochondrial dysfunction with the addition of FCCP/oligomycin. The OCR/ECAR ratio decreased significantly when FCCP/oligomycin was added (p < 0.001). This result is in line with a previous report showing that glycolytic rates increased after 1 h of oligomycin treatment in different cancer cell lines^34^. Only SCC25 cells showed no decrease in the OCR/ECAR ratio; however, xCT expression increased substantially (Suppl. Fig. 2). Wang and coauthors also reported increased xCT expression when cells were treated with oligomycin and antimycin A, which was linked to proliferation and malignant progression^24^.

To confirm that the cells were all capable of epithelial mesenchymal transition, we examined the EMT response to TGF-β1, a canonical trigger of EMT^12,17^. EMT was determined by the gene expression of SNAI2, a master regulator of EMT in HNSCC, and the expression of vimentin, a mesenchymal marker not normally expressed in epithelial cells. In addition, the double positivity of vimentin and cytokeratin, a hallmark of EMT, was measured by flow cytometry and EMT-typical morphologic features were examined by holotomographic microscopy. TGF-β1 caused EMT in all cell lines and with all detection methods. Although not significant, holotomographic microscopy also revealed EMT induction after TGF-β1 stimulation. This finding indicates that the cell lines used were capable of undergoing EMT and our results support observations of other studies that suggested the stabilization of a hybrid epithelial/mesenchymal phenotype through SNAI2^36^. Furthermore, the equivocal role of SNAI2 in maintenance of stem cell pluripotency and DNA stability in healthy and malignant cells is underlined^37^ as simultaneous increase in SNAI2 and vimentin expression was only seen in one cell line.

Mitochondrial dysfunction and EMT regulatory pathways influence each other. EMT transcription factors can affect various metabolic pathways^18^. Downregulation of mitochondrial protein expression involved in OXPHOS was associated with enhanced EMT^7^ as well as oligomycin A administration, leading to downregulation of epithelial factors, increased vimentin expression and subsequent invasion and migration^20^. This is in line with our results, where we could see an increased vimentin expression when cells were treated with oligomycin and FCCP.

Besides stress, several other inhibitors of EMT and its metastatic potential were recently reviewed elsewhere. Reduced proliferative potential after complete EMT and loss of epithelial characteristics has been observed in breast cancer cells^38^. In addition, feedback mechanisms, responsible for mesenchymal–epithelial transformation, were found to inhibit EMT in cells with epithelial characteristics^39^. Finally, stimulation with EMT promoting factors such as TGF-ß might require several days before actually inducing EMT^40^. Correlating these aspects with stress induced mitochondrial dysfunction might thus be an interesting subject of further research.

Thus, it can be assumed that mitochondrial dysfunction did indeed occur and that the cells were capable of EMT. However, the EMT response to mitochondrial dysfunction varied from cell line to cell line. An EMT response was consistently detectable in CAL 27 cells. This may be due to several reasons. One possible explanation could be the different cellular background of each cell line. SCC25 cells exhibit increased baseline expression of EMT proteins such as vimentin^41^. UPCI-SCC-03 cells contain wild-type p53, which is known to suppress EMT^42^. Therefore, it would be explanatory that it was not possible to induce EMT via mitochondrial dysfunction in these two cell lines. HN and CAL-27 cells contain a p53 mutation^43^. However, they cannot be directly compared because HN cells were isolated from a metastatic tumor, which is different from cell lines isolated from primary tumors, such as CAL-27 cells. Therefore, HN cells might have been exposed to other types of cellular stress before and therefore, we were unable to induce EMT with FCCP and oligomycin.

In summary, the study suggests that given the marked heterogeneity of head and neck carcinomas, EMT induced by mitochondrial dysfunction may occur in different clones of HNC in vivo. However, EMT does not appear to be a major or common mechanism of the cancer-promoting effects of mitochondrial dysfunction in HNC. Notwithstanding, other forms of cellular stress, such as hypoxic, nutritional, and heat stress, can trigger EMT in head and neck cancer^44–46^.

## Supplementary Information

Supplementary Information.

## Data Availability

The datasets used and/or analysed during the current study are available from the corresponding author on reasonable request.

## Abbreviations

EMT: Epithelial-mesenchymal transition
TGF-ß: Transforming growth factor beta
OXPHOS: Oxidative phosphorylation
HNSCC: Head and neck squamous cell carcinoma
xCT: Cystine/glutamate transporter
FCCP: Carbonyl cyanide-4-(trifluoromethoxy) phenylhydrazone
OCR: Oxygen consumption rate
ECAR: Extracellular acidification rate
FM: Fibroblast-like-morphology

## References

[1] Porporato PE, Filigheddu N, Pedro JMB, Kroemer G, Galluzzi L (2018). Mitochondrial metabolism and cancer. Cell Res..

[2] Luo Y, Ma J, Lu W (2020). The significance of mitochondrial dysfunction in cancer. Int. J. Mol. Sci..

[3] Vyas S, Zaganjor E, Haigis MC (2016). Mitochondria and cancer. Cell.

[4] Yang J, Ren B, Yang G, Wang H, Chen G, You L, Zhang T, Zhao Y (2020). The enhancement of glycolysis regulates pancreatic cancer metastasis. Cell Mol. Life Sci..

[5] Hanahan D, Weinberg RA (2011). Hallmarks of cancer: The next generation. Cell.

[6] Porporato PE, Payen VL, Baselet B, Sonveaux P (2016). Metabolic changes associated with tumor metastasis, part 2: Mitochondria, lipid and amino acid metabolism. Cell Mol. Life Sci..

[7] Guerra F, Guaragnella N, Arbini AA, Bucci C, Giannattasio S, Moro L (2017). Mitochondrial dysfunction: A novel potential driver of epithelial-to-mesenchymal transition in cancer. Front. Oncol..

[8] Dudás J, Ladányi A, Ingruber J, Steinbichler TB, Riechelmann H (2020). Epithelial to mesenchymal transition: A mechanism that fuels cancer radio/chemoresistance. Cells.

[9] Dongre A, Weinberg RA (2019). New insights into the mechanisms of epithelial-mesenchymal transition and implications for cancer. Nat. Rev. Mol. Cell Biol..

[10] Thiery JP, Acloque H, Huang RY, Nieto MA (2009). Epithelial-mesenchymal transitions in development and disease. Cell.

[11] Cook DP, Vanderhyden BC (2020). Context specificity of the EMT transcriptional response. Nat. Commun..

[12] Ingruber J, Savic D, Steinbichler TB, Sprung S, Fleischer F, Glueckert R, Schweigl G, Skvortsova I-I, Riechelmann H, Dudás J (2021). KLF4, slug and EMT in head and neck squamous cell carcinoma. Cells.

[13] Steinbichler TB, Dudas J, Ingruber J, Glueckert R, Sprung S, Fleischer F, Cidlinsky N, Dejaco D, Kofler B, Giotakis AI (2020). Slug is a surrogate marker of epithelial to mesenchymal transition (EMT) in head and neck cancer. J. Clin. Med..

[14] Puisieux A, Brabletz T, Caramel J (2014). Oncogenic roles of EMT-inducing transcription factors. Nat. Cell Biol..

[15] Ghulam J, Stuerken C, Wicklein D, Pries R, Wollenberg B, Schumacher UDO (2019). Immunohistochemical analysis of transcription factors and markers of epithelial–mesenchymal transition (EMT) in human tumors. Anticancer Res..

[16] Buonato JM, Lan IS, Lazzara MJ (2015). EGF augments TGFbeta-induced epithelial-mesenchymal transition by promoting SHP2 binding to GAB1. J. Cell Sci..

[17] Xu J, Lamouille S, Derynck R (2009). TGF-β-induced epithelial to mesenchymal transition. Cell Res..

[18] Georgakopoulos-Soares I, Chartoumpekis DV, Kyriazopoulou V, Zaravinos A (2020). EMT factors and metabolic pathways in cancer. Front. Oncol..

[19] He X, Zhou A, Lu H, Chen Y, Huang G, Yue X, Zhao P, Wu Y (2013). Suppression of mitochondrial complex I influences cell metastatic properties. PLoS ONE.

[20] Han SY, Jeong YJ, Choi Y, Hwang SK, Bae YS, Chang YC (2018). Mitochondrial dysfunction induces the invasive phenotype, and cell migration and invasion, through the induction of AKT and AMPK pathways in lung cancer cells. Int. J. Mol. Med..

[21] Chow LQM (2020). Head and neck cancer. N. Engl. J. Med..

[22] Canning M, Guo G, Yu M, Myint C, Groves MW, Byrd JK, Cui Y (2019). Heterogeneity of the head and neck squamous cell carcinoma immune landscape and its impact on immunotherapy. Front. Cell Dev. Biol..

[23] Parker JL, Deme JC, Kolokouris D, Kuteyi G, Biggin PC, Lea SM, Newstead S (2021). Molecular basis for redox control by the human cystine/glutamate antiporter system xc−. Nat. Commun..

[24] Wang S-F, Chen M-S, Chou Y-C, Ueng Y-F, Yin P-H, Yeh T-S, Lee H-C (2016). Mitochondrial dysfunction enhances cisplatin resistance in human gastric cancer cells via the ROS-activated GCN2-eIF2α-ATF4-xCT pathway. Oncotarget.

[25] White JS, Weissfeld JL, Ragin CC, Rossie KM, Martin CL, Shuster M, Ishwad CS, Law JC, Myers EN, Johnson JT, Gollin SM (2007). The influence of clinical and demographic risk factors on the establishment of head and neck squamous cell carcinoma cell lines. Oral Oncol..

[26] Kawamata H, Nakashiro K-I, Uchida D, Harada K, Yoshida H, Sato M (1997). Possible contribution of active MMP2 to lymph-node metastasis and secreted cathepsin L to bone invasion of newly established human oral-squamous-cancer cell lines. Int. J. Cancer.

[27] Gioanni J, Fischel J-L, Lambert J-C, Demard F, Mazeau C, Zanghellini E, Ettore F, Formento P, Chauvel P, Lalanne C-M (1988). Two new human tumor cell lines derived from squamous cell carcinomas of the tongue: Establishment, characterization and response to cytotoxic treatment. Eur. J. Cancer Clin. Oncol..

[28] Wang X, Spandidos A, Wang H, Seed B (2012). PrimerBank: A PCR primer database for quantitative gene expression analysis, 2012 update. Nucleic Acids Res..

[29] Wu M, Neilson A, Swift AL, Moran R, Tamagnine J, Parslow D, Armistead S, Lemire K, Orrell J, Teich J (2007). Multiparameter metabolic analysis reveals a close link between attenuated mitochondrial bioenergetic function and enhanced glycolysis dependency in human tumor cells. Am. J. Physiol. Cell Physiol..

[30] Livak KJ, Schmittgen TD (2001). Analysis of relative gene expression data using real-time quantitative PCR and the 2^−ΔΔCT^ method. Methods.

[31] Ingruber J, Dudás J, Savic D, Schweigl G, Steinbichler TB, Greier MDC, Santer M, Carollo S, Trajanoski Z, Riechelmann H (2022). EMT-related transcription factors and protein stabilization mechanisms involvement in cadherin switch of head and neck squamous cell carcinoma. Exp. Cell Res..

[32] Narayanankutty V, Narayanankutty A, Nair A (2019). Heat shock proteins (HSPs): A novel target for cancer metastasis prevention. Curr. Drug Targets.

[33] Giannoni E, Parri M, Chiarugi P (2012). EMT and oxidative stress: A bidirectional interplay affecting tumor malignancy. Antioxid. Redox Signal.

[34] Hao W, Chang CP, Tsao CC, Xu J (2010). Oligomycin-induced bioenergetic adaptation in cancer cells with heterogeneous bioenergetic organization. J. Biol. Chem..

[35] Klussmann JP (2017). Head and neck cancer—New insights into a heterogeneous disease. Oncol. Res. Treat..

[36] Subbalakshmi AR, Sahoo S, Biswas K, Jolly MK (2022). A computational systems biology approach identifies SLUG as a mediator of partial epithelial-mesenchymal transition (EMT). Cells Tissues Organs.

[37] Sterneck E, Poria DK, Balamurugan K (2020). Slug and E-cadherin: Stealth accomplices?. Front. Mol. Biosci..

[38] Eichelberger, L. *et al*. Maintenance of epithelial traits and resistance to mesenchymal reprogramming promote proliferation in metastatic breast cancer. *BioRxiv*. 10.1101/2020.03.19.998823 (2020).

[39] Jia, W. *et al.* Epigenetic feedback and stochastic partitioning during cell division can drive resistance to EMT. 1949–2553 (2020). 10.18632/oncotarget.27651 PMC7343638 32676163

[40] Brown KA, Aakre ME, Gorska AE, Price JO, Eltom SE, Pietenpol JA, Moses HL (2004). Induction by transforming growth factor-β1 of epithelial to mesenchymal transition is a rare event in vitro. Breast Cancer Res..

[41] Sudha R, Kawachi N, Du P, Nieves E, Belbin TJ, Negassa A, Angeletti RH, Prystowsky MB (2007). Global proteomic analysis distinguishes biologic differences in head and neck squamous carcinoma. Lab. Investig..

[42] Ren D, Wang M, Guo W, Zhao X, Tu X, Huang S, Zou X, Peng X (2013). Wild-type p53 suppresses the epithelial-mesenchymal transition and stemness in PC-3 prostate cancer cells by modulating miR145. Int. J. Oncol..

[43] Ghosh S, Bhattacharjee M, Jana NK (2022). Gene regulation by p53 in human cancer system. Asian Pac. J. Cancer Biol..

[44] Joseph JP, Harishankar MK, Pillai AA, Devi A (2018). Hypoxia induced EMT: A review on the mechanism of tumor progression and metastasis in OSCC. Oral Oncol..

[45] Gayan, S., Teli, A., Nair, A. & Dey, T. Nutritional stress alone can control in vitro tumor formation and its invasive nature. *BioRxiv*. 2020:2020.2002.2017.952234.

[46] Seclì L, Fusella F, Avalle L, Brancaccio M (2021). The dark-side of the outside: How extracellular heat shock proteins promote cancer. Cell. Mol. Life Sci..

