# Supplementary material for: Mitochondrial dysfunction and epithelial to mesenchymal transition in head neck cancer cell lines
Source: Sci Rep. 2022 Aug 2;12:13255. doi: 10.1038/s41598-022-16829-5 (PMC9345891; doi:10.1038/s41598-022-16829-5)
Supplement: Supplementary file 1 — Supplementary Information. [file 41598_2022_16829_MOESM1_ESM.docx]

# Supplement

## Supplement 1

**FCCP/oligomycin mixture for one XFp cartridge**

FCCP was suspended with 288 μL XFp assay medium and oligomycin with 252 μL, to reach a final concentration of 50mM. In an Eppendorf tube 180 μL of XFp assay medium was added, then 60 μL of oligomycin stock and finally 60 μL of FCCP stock. 20 μL of FCCP/oligomycin were added to each port of an XFp Seahorse sensor cartridge lid (#103022-100, Agilent Bioscience, Sta. Clara, USA), where it was then injected into the wells at specific time points in the XFp Seahorse Analyzer.

## Supplement 2

**Seahorse XFp Analyzer parameters normalization**

For normalization, medium was removed from all wells, and 30 μL of a 1:1000 HOECHST 33342 (Immuno Chemistry Technologies, Bloomington, US) and PBS (Fresenius Kabi GmbH, Bad Homburg, Germany) mix was added. The plate was incubated for around 10 min in the dark and then counted with the Lionheart™ FX Automated Live Cell Imager (BioTek, Bad Friedrichshall, Germany). After cell counting, HOECHST and XFp Medium were replaced by DMEM/F12 (containing 0.3% FBS) with or without additional 1 ng/mL TGF-β1 in all wells.

## Supplement 3

**FCCP/oligomycin for xCT experiments**

For xCT measurements, cells were incubated and treated with the same amount of FCCP/oligomycin as in the extracellular flux analysis part above. Therefore, a Carbonylcyanid-4-(trifluormethoxy)phenylhydrazon (FCCP) stock solution (254.17 g/mol) (#C2920-10MG, Sigma Aldrich, Darmstadt, Germany) was diluted with 3,8 mL 100% ethanol and 0,2 mL sterile water. An Oligomycin stock solution (#O4876-5MG, Sigma Aldrich, Darmstadt, Germany) was diluted with 630 μL of 100% ethanol. The two 10mM stock solutions were diluted 1:10 with the medium used for the cells and mixed. One μL of FCCP/oligomycin mix was added per mL medium. After four days of treatments and incubation, medium and cells were harvested and xCT expression was measured using PCR (see below).

**Supplementary tables**

## Suppl. table 1

PCR primer sequences for xCT (SLC7A11), vimentin, SNAI 2 (SNAI 2) and the housekeeping gene GAPDH.

| **Gene** | **Forward primer** | **Reverse primer** |
| --- | --- | --- |
| GAPDH | TGCACCACCAACTGCTTAGC | GGCATGGACTGTGGTCATGAG |
| Vimentin [VIM] | TGCCGTTGAAGCTGCTAACTA | CCAGAGGGAGTGAATCCAGATTA |
| xCT [SLC7A11] | GCGTGGGCATGTCTCTGAC | GCTGGTAATGGACCAAAGACTTC |
| SNAI 2 | ACATAAGCAGCTGCACTGCG | ATGGGTCTGCAGATGAGCCC |

## Suppl. table 2

For flow cytometry antibody reactions (one was set for 2 x10^6^ cells) were completed with direct conjugated antibodies in the indicated quantities per reaction.

| **Isotype controls** | **Conjugate** |
| --- | --- |
| 1 μl mouse IgG1 | FITC (Invitrogen) |
| 1 μl mouse IgG1 | PE (Exbio) |
|  |  |
| **Full stainings** | **Conjugate** |
| 5 μl anti-vimentin mouse IgG1 | PE (BD Pharmingen) |
| 10 μl anti-pan-cytokeratin mouse IgG1 | FITC (Beckman Coulter) |

## Suppl. table 3

List of relative OCR/ECAR ratio values in response to FCCP/oligomycin administration (Estimated marginal means ±SEM).

|  | **OCR/ECAR ratio** | |  |
| --- | --- | --- | --- |
| **Cell lines** | **without FCCP/oligomycin** | **with FCCP/oligomycin** | **p-value** |
| SCC25 | 2.1 ± 0.3 | 2.5 ± 0.2 | 0.386 |
| SCC03 | 1.6 ± 0.21 | 1.0 ± 0.1 | 0.017 |
| HN | 4.3 ± 0.6 | 2.4 ± 0.32 | 0.005 |
| CAL-27 | 4.3 ± 0.6 | 2.2 ± 0.3 | <0.001 |

## Suppl. table 4

Relative xCT expression in 4 HNSCC cell lines in response to FCCP/oligomycin (Estimated marginal means ±SEM).

|  | **Relative xCT expression** | |  |
| --- | --- | --- | --- |
| **Cell lines** | **without FCCP/oligomycin** | **with FCCP/oligomycin** | **p-value** |
| SCC25 | 2.4 ± 0.4 | 20.1 ± 3.4 | 0.386 |
| SCC03 | 27.1 ± 4.5 | 70.9 ± 12.1 | 0.017 |
| HN | 3.6 ± 0.6 | 23.7 ± 4.0 | 0.005 |
| CAL-27 | 1.21 ± 0.2 | 8.9 ± 1.5 | <0.001 |

## Suppl. table 5

Relative SNAI2 and vimentin expression in 4 HNSCC cell lines, in response to TGF-β1 addition (Estimated marginal means ± SEM).

|  | **Relative SNAI2 expression** | |  | **Relative vimentin expression** | |  | **Relative % of vimentin-cytokeratin double positivity** | | |
| --- | --- | --- | --- | --- | --- | --- | --- | --- | --- |
| Cell line | without TGF-β1 | with TGF-β1 | p-value | without TGF-β1 | with TGF-β1 | p-value | without TGF-β1 | with TGF-β1 | p-value |
| SCC25 | 11.9 ± 2.1 | 26.3 ± 4.7 | 0.005 | 1.1 ± 0.2 | 8.4 ± 2.0 | <0.001 | 56.8 ± 8.3 | 69.3 ± 10 | 0.339 |
| SCC03 | 11.8 ± 2.1 | 18.0 ± 3.2 | 0.108 | 0.03 ± 0.2 | 0.2 ± 0.003 | <0.001 | 9.4 ± 1,3 | 12.6 ± 1.8 | 0.160 |
| HN | 7.6 ± 1.3 | 17.7 ± 3.1 | 0.003 | 0.02 ± 0.01 | 0.02 ± 0.005 | 0.479 | 6.8 ± 0.9 | 7.7 ±1.1 | 0.541 |
| CAL-27 | 2.3 ± 0.4 | 5.0 ± 0.9 | 0.007 | 0.003 ± 0.0 | 0.03 ± 0.01 | 0.004 | 53.7 ± 7.8 | 43.5 ± 6.3 | 0.311 |

## Suppl. table 6

Values of EMT holotomographic scoring in response to TGF-β1 administration (range of possible scores from 0 to 9; Fisher-Freeman-Halton p=0.10)

| **EMT Holotomographic scoring * With/without TGF-β1 Cross tabulation** | | | | |
| --- | --- | --- | --- | --- |
| Count | | | | |
|  | | With/without TGF-β1 | | Total |
|  |  | without TGF-β1 | with TGF-β1 |  |
| EMT-score | 2 | **5** | **1** | **6** |
|  | 3 | **7** | **7** | **14** |
|  | 4 | **3** | **8** | **11** |
|  | 5 | **1** | **0** | **1** |
| Total | | **16** | **16** | **32** |

# Supplementary figures

## Suppl. Figure 1


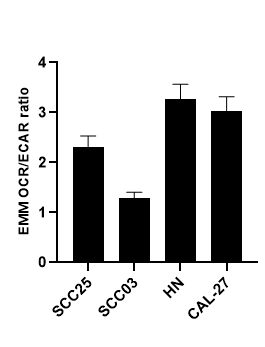

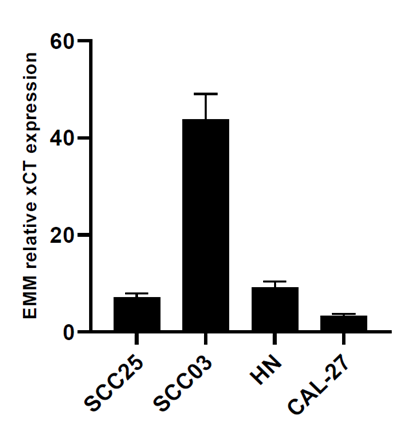


**A**

**B**

Baseline OCR/ECAR ratio A) and xCT expression B) in 4 HNSCC cell lines (EMM: Estimated marginal mean; Bars: SEM). Note the inverse correlation between OCR/ECAR ratio and xCT expression.

## Suppl. Figure 2


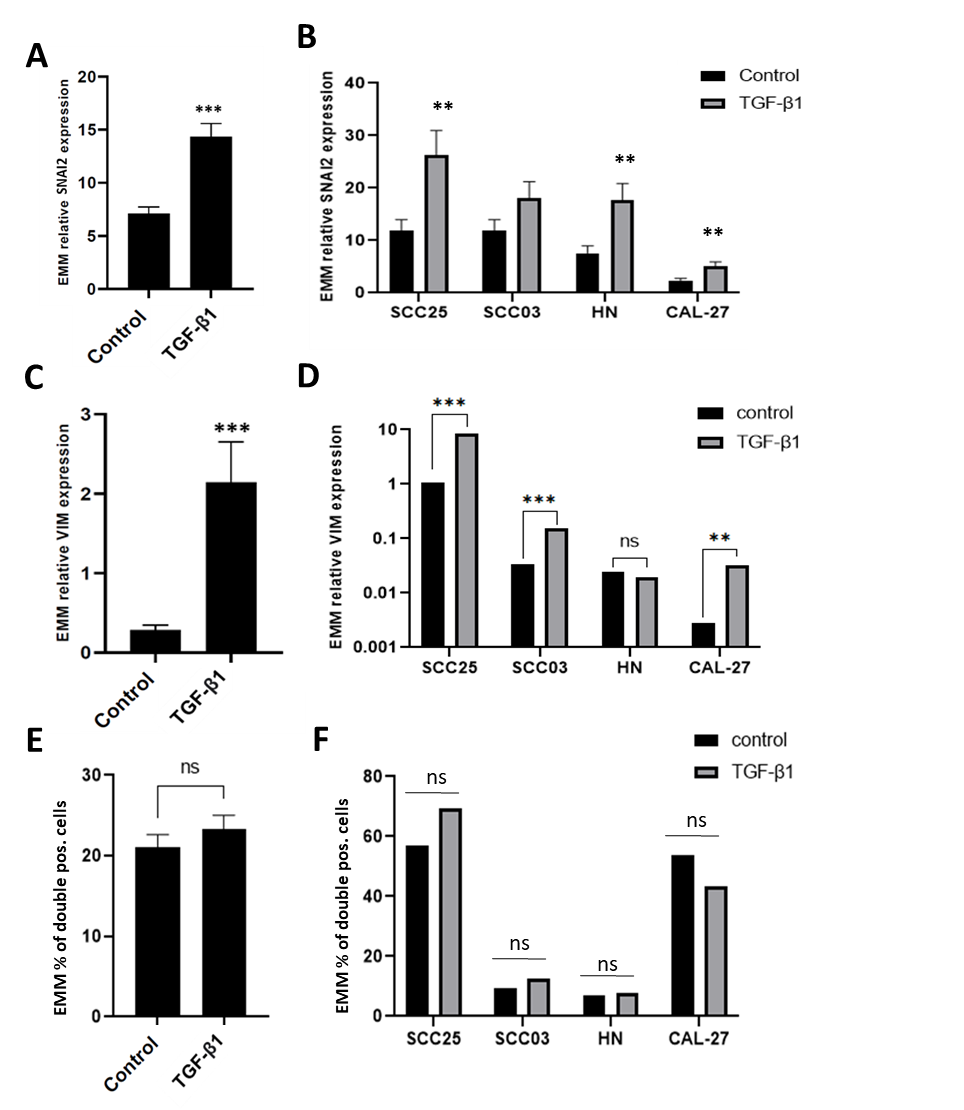


Relative SNAI2 (A and B), vimentin expression (C and D) and vimentin/cytokeratin double positivity (E and F) of 4 HNC cell lines in response to TGF-β1 (EMM: Estimated marginal mean; Bars: SEM; *p<0.05, ** p<0.01, *** p<0.001, ns - not significant).
